# Chromosomal Deletion Involving ANKRD26 Leads to Expression of a Fusion Protein Responsible for ANKRD26-Related Thrombocytopenia

**DOI:** 10.3390/ijms26157330

**Published:** 2025-07-29

**Authors:** Gianluca Dell’Orso, Tommaso Passarella, Serena Cappato, Enrico Cappelli, Stefano Regis, Massimo Maffei, Matilde Balbi, Silvia Ravera, Daniela Di Martino, Silvia Viaggi, Sabrina Davì, Fabio Corsolini, Maria Carla Giarratana, Luca Arcuri, Eugenia Mariani, Riccardo Morini, Erika Massaccesi, Daniela Guardo, Michaela Calvillo, Elena Palmisani, Domenico Coviello, Francesca Fioredda, Carlo Dufour, Renata Bocciardi, Maurizio Miano

**Affiliations:** 1 Hematology Unit, IRCCS Istituto Giannina Gaslini, 16147 Genoa, Italy; tommasopassarella@gmail.com (T.P.); danieladimartino@gaslini.org (D.D.M.); fabiocorsolini@gaslini.org (F.C.); mariacarlagiarratana@gaslini.org (M.C.G.); lucaarcuri@gaslini.org (L.A.); eugeniamariani@gaslini.org (E.M.); erikamassaccesi@gaslini.org (E.M.); danielaguardo@gaslini.org (D.G.); michaelacalvillo@gaslini.org (M.C.); elenapalmisani@gaslini.org (E.P.); francescafioredda@gaslini.org (F.F.); carlodufour@gaslini.org (C.D.); mauriziomiano@gaslini.org (M.M.); 2 Unit of Medical Genetics, IRCCS Istituto Giannina Gaslini, 16147 Genoa, Italy; serenacappato@gaslini.org (S.C.); renatabocciardi@gaslini.org (R.B.); 3 Laboratory of Clinical and Experimental Immunology, IRCCS Istituto Giannina Gaslini, 16147 Genoa, Italy; stefanoregis@gaslini.org; 4 Laboratory of Human Genetics, IRCCS Istituto Giannina Gaslini, 16147 Genoa, Italy; massimomaffei@gaslini.org (M.M.); silviaviaggi@gaslini.org (S.V.); sabrinadavi@gaslini.org (S.D.); domenicocoviello@gaslini.org (D.C.); 5 Experimental Medicine Department, University of Genoa, 16132 Genoa, Italysilvia.ravera@unige.it (S.R.); 6 IRCCS Policlinico San Martino, 16132 Genoa, Italy; 7 DISTAV (Department of Earth, Environment and Life Sciences), University of Genova, 16132 Genoa, Italy; 8 DINOGMI (Department of Neuroscience, Rehabilitation, Ophthalmology, Genetics, Maternal and Child Health), University of Genoa, 16126 Genoa, Italy

**Keywords:** ANKRD26, inherited thrombocytopenia, chromosomal deletion, fusion protein

## Abstract

ANKRD26-related thrombocytopenia (ANKRD26-RT) is characterized by lifelong mild to moderate thrombocytopenia. Patients suffer from an increased susceptibility to acute or chronic myeloid leukemia, myelodysplastic syndrome, or chronic lymphocytic leukemia. We described here a patient with inherited thrombocytopenia initially misdiagnosed as immune thrombocytopenic purpura. A chromosomal deletion involving the *ANKRD26* gene was identified. Gene and protein expression analyses suggest an alternative pathogenic mechanism of altered megakaryopoiesis: the synthesis of a chimeric protein with aberrant expression due to the unregulated action of a promoter from a gene located upstream of *ANKRD26*. This study highlights the importance of advanced genetic testing and functional analysis of patients’ primary cells in the case of the detection of previously unrecognized structural variants in order to understand pathogenic mechanisms. These investigations provided a definitive diagnosis for the patient and facilitated the development of a tailored clinical management strategy, especially concerning the potential for myeloid transformation.

## 1. Introduction

The diagnosis of inherited thrombocytopenia (IT) is based on the association of reduced platelet count with different functional and morphological platelet features and extra-hematological phenotypes [1]. The identification of IT can be challenging due to variable phenotypic penetrance and heterogeneous clinical presentation [2]. In the pediatric population, it can be misdiagnosed with the more common immune thrombocytopenic purpura (ITP). In addition to the most typical syndromic or non-syndromic ITs, characterized by a related bleeding risk or specific structural/functional alterations in platelets, a number of novel congenital thrombocytopenias have been recently described, showing less significant thrombocytopenia and bleeding features. In these cases, despite a milder phenotype, patients can be at risk of developing hematological malignancies as well [3]. The number of congenital IT has been increasing over the years, and so far, at least 40 genes have been described [4].

ANKRD26-related thrombocytopenia (ANKRD26-RT), also identified as Thrombocytopenia 2 (THC2, OMIM #188000), is defined as a non-syndromic autosomal dominant disorder, characterized by variable bleeding features associated with lifelong mild to moderate thrombocytopenia with normal mean platelet volume [5]. The clinical phenotype is secondary to impaired bone marrow megakaryopoiesis with an increase in small and hypolobulated platelet precursors, often resembling myelodysplasia (MDS) [6]. ANKRD26-RT patients suffer from an increased susceptibility to hematological malignancy: an 8% lifetime risk of developing acute myeloid leukemia (AML), MDS, chronic myeloid leukemia (CML), or chronic lymphocytic leukemia (CLL) has been estimated [2]. Due to the bleeding features and the frequent wrong diagnosis of ITP in children, in the literature, unsuccessful therapeutic approaches with immunosuppressants have been reported [7].

*ANKRD26* is a gene mapping to chromosome 10p11.1-p12. The so-called *TCH2* locus was identified in 1999 in a family with autosomal dominant thrombocytopenia [8], and several studies aimed to define the specific gene responsible for the clinical phenotype, considering *MASTL* [9] and then *ACBD5* as candidate genes [10]. The role of ANKRD26 in the pathogenesis of IT was first described in 2011 [11], and it was usually related to single-nucleotide variants (SNV) in the 5′-untranslated region (5′-UTR) [5,11] of the gene, leading to a disrupted binding of transcriptional factors RUNX1 and FLI1 and altered regulation of gene expression during megakaryopoiesis. However, recent findings suggest different pathogenic mechanisms potentially involved in altered megakaryopoiesis. A complex paired-duplication inversion reported by Wahlster resulted in the generation of a fusion transcript involving the *WAC* promoter and exon 1 and *ANKRD26* from exons 10 to 34 [12]. A *WAC-ANKRD26* fusion transcript led to the upregulation of *ANKRD26* mRNA levels (approximately 50 folds), comprising the exons included in the structural rearrangement and an increase in ERK phosphorylation. This finding was considered a functional demonstration of the role of a truncated form of ANKRD26 to increase MAPK activation of the thrombopoietin (TPO) receptor. Recently, a deletion of the 10p12.1 chromosome in the *THC2* locus involving exons 1 to 4 of *ANKRD26* was described in a family of patients affected by thrombocytopenia [13]. Authors speculated about a different pathogenic mechanism associated with the deletion in this family, such as a loss of function, a dosage-sensitive effect of the involved region, or a role of another gene included in this region [13], but no molecular data were reported to support their assumptions.

### Case Report

**Figure 1:**
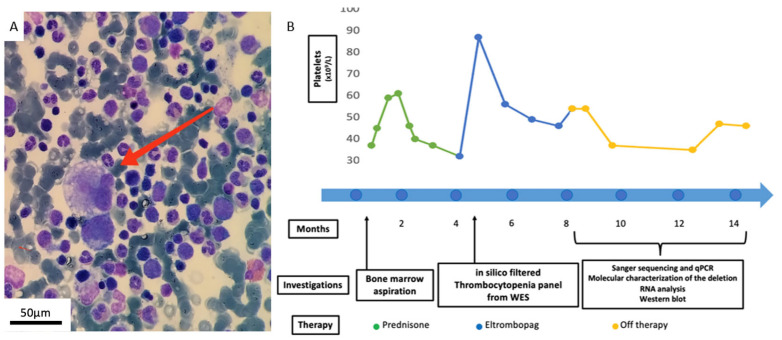
(**A**) Bone marrow morphological features show an altered megakaryocyte maturation with nuclear hypolobulation and peripheral vacuolization (red arrow, magnification 60×). (**B**) Platelet count, investigations, and treatment outline the patient’s clinical history.

A boy, born to healthy non-consanguineous parents, was admitted to the Emergency Room due to the sudden onset of isolated severe thrombocytopenia (platelets 6 × 10^9^/L), preceded by a self-limiting infectious event characterized by fever and vomiting. The patient received an initial diagnosis of ITP and was treated with intravenous immunoglobulin (IVIG) in repeated cycles due to loss of response, and later with high-dose prednisone, later tapered over 12 weeks, with an unsuccessful response at 34 × 10^9^/L. Bone marrow examination revealed active proliferation of megakaryocytes, characterized by nuclear hypolobulation and peripheral vacuolization (Figure 1A). The patient received second-line treatment with the thrombopoietin receptor agonist Eltrombopag 25 mg/day, later adjusted according to platelet count, for 5 months. Platelet counts reached 87 × 10^9^/L, 56 × 10^9^/L, and 54 × 10^9^/L at 4, 8, and 20 weeks of treatment, respectively, showing therefore only a brief short-term response despite increased dose. At Eltrombopag discontinuation, the platelet count fluctuated from 30 to 47 × 10^9^/L, without a significant worsening of blood counts compared to the previous weeks and no additional severe mucocutaneous hemorrhagic events. Figure 1B summarizes platelet counts over time despite different treatment strategies and the diagnostic evaluations performed.

Due to the unsatisfactory response to treatment and morphological abnormalities in bone marrow, an in silico-filtered Thrombocytopenia panel (see Table S1) from WES led to the identification of a missense variant c.500G > A p.(Arg167Gln; dbSNP, rs772757150) in the *ARPC1B* gene (NM_005720.4) and a deletion of approximately 160kb of the short arm of chromosome 10.

The heterozygous *ARPC1B* variant of paternal origin was considered a variant of uncertain significance (VUS) according to ACMG criteria (PM2, PP3) [14], and it was not related to clinical features, as ARPC1B deficiency is characterized by autosomal recessive inheritance.

**Figure 2:**
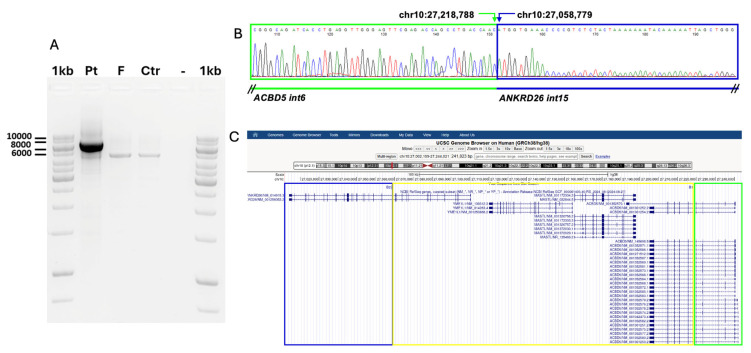
Characterization of the deletion’s junction fragment at the genomic level. (**A**) Long-PCR with oligonucleotides specific for exon 6 of ACBD5 and for exon 16 of ANKRD26 genes showing the presence of a specific amplification product (upper band) of approx. 6518 bp only with DNA of the patient (Pt) and not of the unaffected father (F) nor of a control individual (Ctr). A lower band correspoding to a non specific product is detectable in all the samples. (**B**) Chromatograms showing the sequence at the junction between ACBD5 and ANKRD26 genes at the genomic level, indicated from 5′ to 3′. (**C**) Genomic window (GRCh/hg38,chr10:27,002,199-27,244,021) obtained from the the UCSC Genome browser from the ANKRD26 to the ACBD5 gene indicating the position of the deletion (yellow rectangle) of approx. 160 kb and the two breakpoints B1 (chr10:27,218,788) in ANKRD26 intron 15, and B2 (chr10:27,218,788) in ACBD5 intron 6. The position of the genes appears to be inversed compared to the panel B, because they are located on the minus strand on the short arm of the chromosome 10. 1kb, molecular weight marker; Pt, patient; F, father; Ctr, control individual; -, no template control for PCR.

The chromosome 10 deletion included *ANKRD26* (from exon 1 to 15), *YME1L1* (whole gene), *MASTL* (whole gene), and *ACBD5* (from exon 7 to 13). The family trio showed that the rearrangement was of de novo origin (see Figure 2A). qPCR validation was performed by using two specific amplicons for ANKRD26 and two for MASTL (Figure S2, Table S2).

Inspired by the work of Wahlster et al. [12], we performed an analysis aimed at linking a deletion detected in a patient to a similar molecular effect on the generation of a fusion protein.

## 2. Results

### 2.1. Molecular Characterization of the Deletion and Identification of an ACBD5/ANKRD26 Fusion Transcript

By a long-PCR approach, we could amplify a junction fragment of approximately 6518 bp in the patient’s DNA, which was absent in the father’s and control DNA (Figure 2A). The two deletion breakpoints were mapped in *ANKRD26* intron 15 (chr10:27,058,779) and *ACBD5* intron 7 (chr10:27,218,788), respectively, with a DNA loss of 160 kb. Since both genes show the same orientation on the minus strand of chromosome 10, and the last intact exon for *ACBD5* and the first preserved exon for *ANKRD26* are in-frame within their respective coding sequence and with each other, we hypothesized the possible formation of a fusion transcript. To verify this hypothesis, we used specific oligonucleotides to amplify complementary DNA (cDNA) retrotranscribed from total RNA obtained from the whole blood of the patient, his father, and a control individual. A PCR product of the expected size was detectable only in the patient’s cDNA and not in the father’s nor the control’s. Sequence analysis confirmed that the PCR product was specific for an *ACBD5-ANKRD26* fusion transcript, with the expression driven by the regulatory regions of the *ACBD5* gene, which also provides the first 6 coding exons. The *ANKRD26* gene contributes to the last coding exons from 16 to 34 (Figure 2B). We also quantified the overall abundance of the *ACBD5* and *ANKRD26* mRNAs compared to the expression of different allele-specific transcripts by Quantitative Polymerase Chain Reaction (qPCR). The expression of ACBD5 was higher than that of ANKRD26 and similar to that of the fusion transcript, detected only in the patient. For this reason, the overall amount of the *ANKRD26* mRNAs deriving from both the wild type and the deleted allele appeared to be more abundant compared to what was observed in the patient’s father (Figure 2C).

### 2.2. ACBD5/ANKRD26 Fusion Transcript Is Translated into a Chimeric Protein

**Figure 3:**
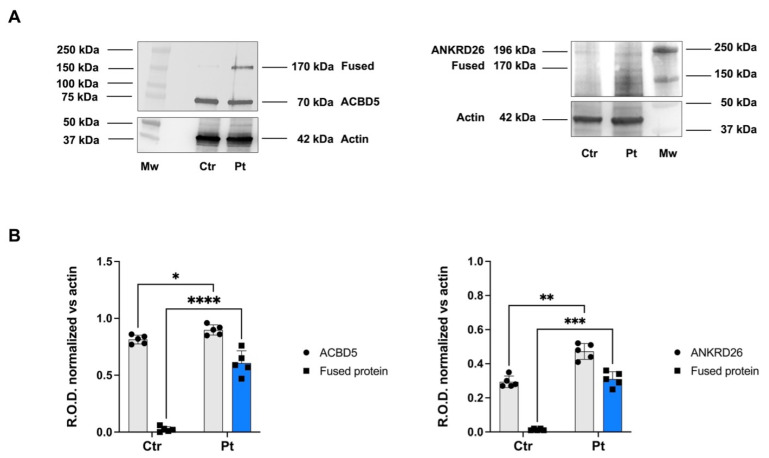
The ACBD5/ANKRD26 fusion transcript is translated into a chimeric protein. (**A**) Representative WB of the ACBD5/ANKRD26 fusion protein. Ctr, lysates from a control individual; Pt, lysates from the patient; Mw, molecular weight markers. (**B**) Densitometric analysis of ACBD5, ANKRD26, and fused protein expression as Relative Optical density (R.O.D.) normalized versus the actin signal, used as housekeeping. Data in panel B are expressed as mean ± SD and are representative of five experiments. *, **, ***, and **** indicate a significant difference for *p* < 0.05, 0.01, 0.001, or 0.0001, respectively.

We then verified that the fusion transcript could be translated into a chimeric protein carrying the NH3-ter of the ACBD5 protein fused to the COOH-half of ANKRD26. Western blot (WB) analysis of total cell lysates from blood cells identified a specific band around 170 kDa, detectable by both anti-ACBD5 and anti-ANKRD26 antibodies, corresponding to the fusion protein. The wild-type counterpart of each protein was also detected by the corresponding antibody (Figure 3A). Densitometric analysis of ACBD5 and ANKRD26 expression showed a significant difference in the level of expression of the protein fused between the cell lysate of the patient and a control individual (Figure 3B), confirming the higher expression of ACBD5 and ANKRD26 in patients compared to the control sample.

## 3. Discussion

ANKRD26 is a 192kDa protein, interacting with the plasma membrane and mediating protein-protein interactions for cytoskeleton and signaling pathways, such as TPO/MPL downstream pathways, JAK/STAT, PI3K, and MAPK/ERK [2,3,5]. ANKRD26 is highly expressed in hematopoietic stem cells, with high mRNA levels in CD34+ and immature megakaryocytes, but it is downregulated during megakaryopoiesis [15]. Mature megakaryocytes CD41+ CD42- —and platelets in AKRD26-RT patients show a persistent expression of the ANKRD26 in contrast to megakaryocytes from wild-type patients [15]. Experimental models demonstrated that overexpressed ANKRD26 accumulates at the cell membrane, disrupting MPL trafficking and JAK/STAT, PI3K, and MAPK/ERK downstream signaling [3,15]. A decrease in MAPK/ERK signaling during normal megakaryocytopoiesis is necessary for proplatelet formation [15]; therefore, ANKRD26 overexpression in ANKRD26-RT and MAPK/ERK hyperactivation alter megakaryocyte maturation. In accordance, the previous works demonstrated that pharmacological inhibition of this pathway restored proplatelet formation [15,16].

Genetic alterations resulting in increased ANKRD26 expression have been associated with ANKRD26-RT. SNVs affecting a highly conserved sequence of the *ANKRD26* 5′UTR have been shown to increase gene expression through the alteration of the binding sites for transcriptional factors RUNX1 and FLI1 [17,18], known to downregulate *ANKRD26* expression during megakaryocyte maturation.

In addition, recent advances have led to the identification of structural alterations responsible for abnormal familial thrombocytopenia due to aberrant *ANKRD26* expression. Specifically, Wahlster et al. reported a complex paired-duplication inversion that resulted in a fusion transcript involving the *WAC* promoter and exon 1, as well as *ANKRD26* from exons 10 to 34 [12]. As a consequence, a *WAC-ANKRD26* fusion transcript and upregulation of *ANKRD26* mRNA levels (approximately 50 folds), comprising the exons included in the structural rearrangement RNA sequencing, were found in peripheral blood mononuclear cells. *WAC* mRNA in normal conditions is ubiquitously expressed in the hematopoietic system at much higher levels than *ANKRD26*. Therefore, the *WAC* promoter led to overexpression of the preserved *ANKRD26* C-terminal region downstream of the rearrangement [12] during the whole megakaryocyte maturation. This was considered responsible for the thrombocytopenia in the absence of 5′UTR variants, as the authors demonstrated increased ERK phosphorylation with either an overexpression of both a full-length or a truncated ANKRD26, with missing ankyrin repats and preserved coiled-coil domains, through lentiviral injection of cDNA from the complex structural variant into primary human CD34+ HSPCs from healthy donors [12].

In a more recent report describing the deletion of the 10p12.1 chromosome in the *THC2* locus involving exons 1 to 4 of *ANKRD26* in a family of patients affected by thrombocytopenia [13], no molecular insights were provided. The authors speculated about a pathogenic mechanism related to a loss of function, a dosage-sensitivity of the involved region, or a role of another gene comprised in this region [13], but the hypothesis of a fusion transcript between *MPP7* and the *ANKRD26* genes has not been considered nor investigated, thus a gain of function mechanism mediated by the rearrangement cannot be ruled out.

Inspired by the work of Wahlster et al. [12], we conducted an analysis aimed at linking our deletion to a similar molecular effect on the generation of a fusion protein. In our patient, a genomic deletion of 160 kb was not missed by the in silico filtered Thrombocytopenia panel (see Table S1) from Whole Exome Sequencing (WES). The genomic region surrounding the deletion breakpoint in intron 15 of *ANKRD26* and intron 6 of *ACBD5* harbors repeated sequences (AluSc5, Class Alu, Family SINE, chr10:27,057,657–27,057,965 in intron 15 of *ANKRD26*; AluSx, Class Alu, SINE family, chr10:27,058,583–27,058,875 in intron 6 of *ACBD5*), characterized by a modular structure and high sequence homology. The structural features of these genomic elements represent a known factor of increased risk for chromosomal rearrangements, and a similar Alu-mediated mechanism was also considered responsible for the complex rearrangement involving *ANKRD26* reported by Wahlster [12]. We provided a deep molecular characterization of the deletion by isolating the junction fragment and demonstrated the formation of a fusion transcript made by the first coding exons of *ACBD5* and exons from 16 to 34 of *ANKRD26*. The generation of fusion transcripts is a well-known cause of altered gene expression in cancer, but increasing interest is also growing in rare inherited conditions [19]. The mRNA expression is driven by the regulatory regions of the *ACBD5*, a gene that is not regulated in hematopoietic stem cells. As a consequence, this fusion transcript is overexpressed and translated into a chimeric *ACBD5-ANKRD26* protein carrying the intact coil-coiled domains of ANKRD26. According to the finely tuned expression of *ANKRD26* during megakaryopoiesis, the finding of an increased number of small megakaryocytes characterized by nuclear hypolobulation and peripheral vacuolization was in accordance with the hypothesis of an effect of the unregulated chimeric fusion protein on proplatelet maturation. As a limitation of our study, we could not demonstrate an increased ERK phosphorylation. However, experimental data from Wahlster [12] demonstrated that a chimeric fusion protein containing a coiled-coil domain was able to increase ERK phosphorylation.

The clinical history of ANKRD26-RT patients is not predictable, as it is associated with nearly 100% penetrance of thrombocytopenia but variable and incomplete penetrance for malignancy, particularly MDS/AML [20]. Delayed onset and incomplete penetrance also suggest that the *ANKRD26* variant may not be sufficient, and clonal haematopoiesis has been identified in two different *ANKRD26* germline variant carriers [21,22], with the addition of somatic variants in *SF3B1* or *ASXL1* and *KRAS*, respectively. Therefore, according to the genetic diagnosis and functional validation, we tailored a careful surveillance program for the patient, based on annual bone marrow evaluation with morphology, cytogenetics, and genetic analysis based on an NGS panel focused on acquired somatic mutations in genes associated with myeloid transformation [2,5,20,22,23,24,25]. Evidence-based clinical data showed less effectiveness of short-term Eltrombopag and a higher starting dose in ANKRD26-RT compared to other ITs [26], probably due to the constitutional hyperactivation of the MAPK/ERK pathway in ANKRD26-RT [15]. Questions have also been raised about the use of TPO mimetics in patients at risk for malignant transformation or due to the risk of developing marrow fibrosis [3]. But, available data is focused only on short-term use [26]. Accordingly, our patient will receive on-demand TPO-agonist treatment in case of surgical procedures or specific bleeding risks [26,27], in particular mucosal bleeding.

## 4. Conclusions

When considering the genetic diagnosis of an IT but also more generally a rare inherited condition, the risk of missing a genetic diagnosis due to sample type, gene list selection, or technology selection should be considered [19], based on potential different genetic alterations [2]. Different genetic tests, such as Sanger sequencing, WES, whole genome sequencing, long-read genome sequencing, and RNA sequencing, should be integrated together with awareness about their diagnostic power and potential risks of incomplete diagnosis [12]. In our patient, an in silico filtered panel from WES was effective in identifying a potential involvement of ANKRD26, but due to the absence of previous, similar findings in the literature, the integration of different genetic tests and complementary functional assays was essential in supporting the pathogenic hypothesis and defining the molecular alteration. The importance of a definite genetic diagnosis is especially relevant in conditions at risk for malignant transformation. For example, in the case of malignancy related to IT, HSCT after remission induction is recommended [20]. The identification of a predisposition to AML is relevant as it can help in avoiding the selection of a matched family donor carrying the same genetic variant [28,29]. Overall, genetic profiling in patients and donors is recommended in preparation for an HSCT in all MDS/AML, as studies suggest that about 10% of all patients with AML/MDS harbor germline variants [30,31], which can occur de novo, as seen in our patients, or be inherited from an apparently asymptomatic parent carrier.

## 5. Materials and Methods

### 5.1. Whole Exome Sequencing

The genomic DNA of the patient was extracted from peripheral blood using the automatic extractor Symphony (Qiagen, Düsseldorf, Germany). WES was performed on genomic coding regions and exon–intron junctions (5 nucleotides) using the WES_v1: 20,133 genes (SOPHiA Genetics, Lausanne, Switzerland) kit on a NovaSeq 6000 platform (Illumina (San Diego, CA, USA)—Italian Institute of Technology). The patient’s WES data underwent a comprehensive analysis of 41 genes (Table S1) associated with Thrombocytopenia, as suggested by the Human Phenotype Ontology browser (HPO). Data filtration and interpretation were performed using SOPHiA DDM software (https://www.sophiagenetics.com/sophia-ddm/, accessed on 17 July 2025) (SOPHiA Genetics, Lausanne, Switzerland), which included Copy Number Variations (CNV) analysis (Figure S1). The minimum target read depth was 20× with optimal coverage of 99%. Data were filtered for high quality (an alternative allele frequency > 30% rare variants with minor allele frequency and MAF < 0.5% according to the GnomAD database). Our WES is based on reading short reads (fragments) of 150 nucleotides, and the limit of detection for CNVs is at least 3–5 consecutive exons. The reference databases used were the human reference genome hg38 and the Human Gene Mutation Database dbSNP15. We used the Integrative Genomic Viewer (IGV) tool to visualize sequence data and variant calls. The pathogenicity of putative germline variants and residue conservation were evaluated according to the American College of Medical Genetics and Genomics (ACMG) [8] guidelines; the bioinformatic analysis of novel variants used classifications from public databases (PolyPhen-2, SIFT, GERP, and others) was supported by SOPHiA DDM, Varsome, and ClinVar.

### 5.2. Sanger Sequencing and Quantitative Polymerase Chain Reaction

Sanger sequencing was performed in the family trio for validation and segregation analysis of the missense variant (BigDye Cycle Sequencing Kit—Applied Biosystems), with primers available in Table S3. The sequencing results were interpreted using two Sequencing Analysis software programs: SeqScape v2.6 (AppliedBiosystem, Foster City, CA, USA) and Alamut Visual Plus v1.8 (SOPHiA Genetics, Lausanne, Switzerland)

The CNV detected by Sophia was validated by qPCR performed in a Light Cycler 480 (Roche). GAPDH was used as a reference gene for normalization. Each DNA sample was run in triplicate in a 20 µL reaction mixture containing 25 ng of DNA, 0.10 mM of each primer, and 1x SYBR Green PCR MasterMix (Roche). The amplification conditions were as follows: a 5-min preincubation at 95 °C followed by 45 cycles of 10 s at 95 °C, 15 s at 60 °C, and 15 s at 72 °C. The PCR products were subjected to a linear temperature transition from 65 °C to 95 °C at 0.3 °C/s. Light Cycler 480 Software vLCS480 1.5.0.39 (Roche Diagnostic, Basel, Switzerland) was used for analysis by the ΔΔCT method. The hemizygous deletion was determined when the relative copy number value for the specific sample, normalized to the reference sample, was less than 0.7. Primers used for qPCR are available in Table S2.

### 5.3. Molecular Characterization of the Deletion

To identify the deletion’s junction fragment, 100 ng of genomic DNA was used as a template for PCR amplification with specific oligonucleotides, with the forward designed in ACBD6 exon 5 and the reverse in the ANKRD26 exon 18 (sequences available upon request), using the Platinum Taq DNA Polymerase (Invitrogen, Carlsbad, CA, USA), according to the manufacturer’s suggestions.

### 5.4. RNA Isolation and Gene Expression

Peripheral blood mononuclear cells (PBMCs) were isolated using Ficoll Paque Plus (Merck, Darmstadt, Germany). RNA was extracted using the RNeasy Plus mini kit (Qiagen).

To evaluate gene expression, 500 ng of total RNA were reverse-transcribed using the Advantage RT-for-PCR Kit (Takara, Kyoto, Japan), according to the manufacturer’s instructions. Gene expression profiles were evaluated through qPCR using the SYBER Green system (iQ SYBER^®^Green Supermix, Biorad, Hercules, CA, USA). Reactions were set up in duplicate in 20 µL volumes using 5 µL of diluted cDNA with oligonucleotides, allowing quantification of the overall expression of each gene and different alleles (deleted, undeleted, and fusion transcripts). qPCR was performed on the iQ5 instrument (BioRad). The presence of a single specific amplification product was checked by melting curve analysis, and negative controls were presented in all runs performed. All samples were quantified in the same run, and the expression of the indicated transcripts was normalized to the *GAPDH/HPRT* housekeeping genes and given as 2^ΔCt^ values. The sequences of all the oligonucleotides applied are available upon request.

### 5.5. Western Blot

Cells were resuspended in phosphate-buffered saline (PBS) and sonicated for 10 s on ice to prevent warming of the mixture, using the Microson XL Model DU-2000 (Misonix Inc., New York, NY, USA). Based on total protein content estimated using the Bradford method [32], 30 μg of protein was loaded for each sample. After denaturing electrophoresis (SDS-PAGE), performed on 4–20% gradient gels, the nitrocellulose membrane was incubated with anti-ACBD5 (Novus Biologicals (Littleton, CO, USA), part of Bio-Techne | Catalog # NBP1-59820) diluted 1:500 in PBS plus 0.15% Tween (PBSt, Tween was from Roche, Basilea, Switzerland, #11332465001), anti-ANKRD26 (Abcam Catalog #AMab86780, Cambridge, UK) diluted 1:2000 in PBSt, and anti-Actin diluted 1:1000 in PBSt (Invitrogen, Catalog # 15G5A11/E2). Appropriate secondary antibodies (Sigma-Aldrich (St. Louis, MI, USA), Catalog #A0168 and #SAB3700870) were diluted 1:10,000 in PBSt. Bands were evaluated by a chemiluminescence system (Alliance 6.7 WL 20M, UVITEC, Cambridge, UK) using an enhanced chemiluminescence substrate (ECL, BioRad, Catalog #1705061). Band intensity was evaluated with ImageJ, (1.52q version), and ACBD5 and ANKRD26 signals were normalized versus the actin signal.

## Data Availability

Original contributions presented in this study are included in the article/Supplementary Materials. Further inquiries can be directed to the corresponding author(s).

## Supplementary Materials

The following supporting information can be downloaded at: https://www.mdpi.com/article/10.3390/ijms26157330/s1.

## Abbreviations

The following abbreviations are used in this manuscript:

ACMG	American College of Medical Genetics
AML	Acute Myeloid Leukemia
ANKRD26-RT	ANKRD26-related thrombocytopenia
cDNA	complementary DNA
CML	Chronic Myeloid Leukemia
CLL	Chronic Lymphocytic Leukemia
CNV	Copy Number Variation
IT	Inherited Thrombocytopenia
ITP	Immune Thrombocytopenic Purpura
MDS	Myelodisplasia
PBSC	Peripheral Blood Stem Cell
PBS	Phosphate-buffered Saline
qPCR	Quantitative Polymerase Chain Reaction
SNV	Single Nucleotide Variants
TPO	Thrombopoietin
UTR	Untranslated Region
VUS	Variant of Unknown Significance
WB	Western Blot
WES	Whole Exome Sequencing
