# Supplementary material for: Chromosomal Deletion Involving ANKRD26 Leads to Expression of a Fusion Protein Responsible for ANKRD26-Related Thrombocytopenia
Source: Int J Mol Sci. 2025 Jul 29;26(15):7330. doi: 10.3390/ijms26157330 (PMC12347728; doi:10.3390/ijms26157330)
Supplement: Supplementary file 1 [file ijms-26-07330-s001.zip › ijms-3735689-supplementary.pdf]

# Figure S1. Level plots of CNV for the patient

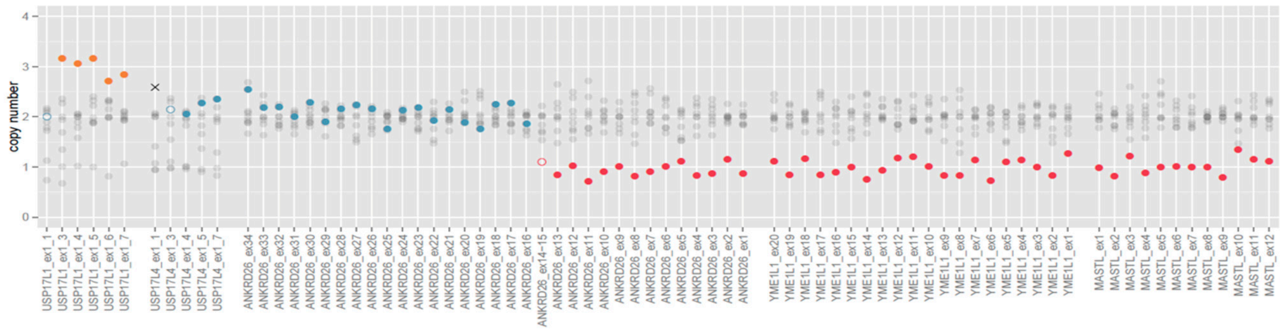


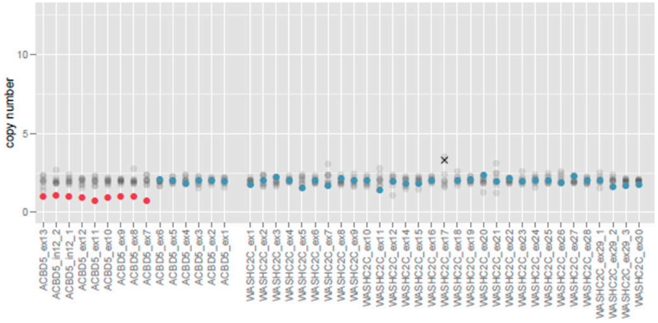


**Level plots of CNV for the patient.**
The plot of normalized coverage levels shown is obtained by software Sophia DDM (pipeline ILL1XG1G4_CNV_exome).
Each plot displays the normalized coverage levels in a given sample (blue, red, and orange dots) compared to the reference coverage levels (grey dots that are samples of the same experiment). On the abscissas the name of genes and specific exon evaluated. Blue dots correspond to target regions without CNVs, red dots to deletions, and orange dots to duplications (insertions). Solid dots are high-confidence CNV predictions, empty dots are medium-confidence predictions, and crosses mark target regions with undetermined CNV status.

# Figure S2. qPCR validation of the deletion identified by WES

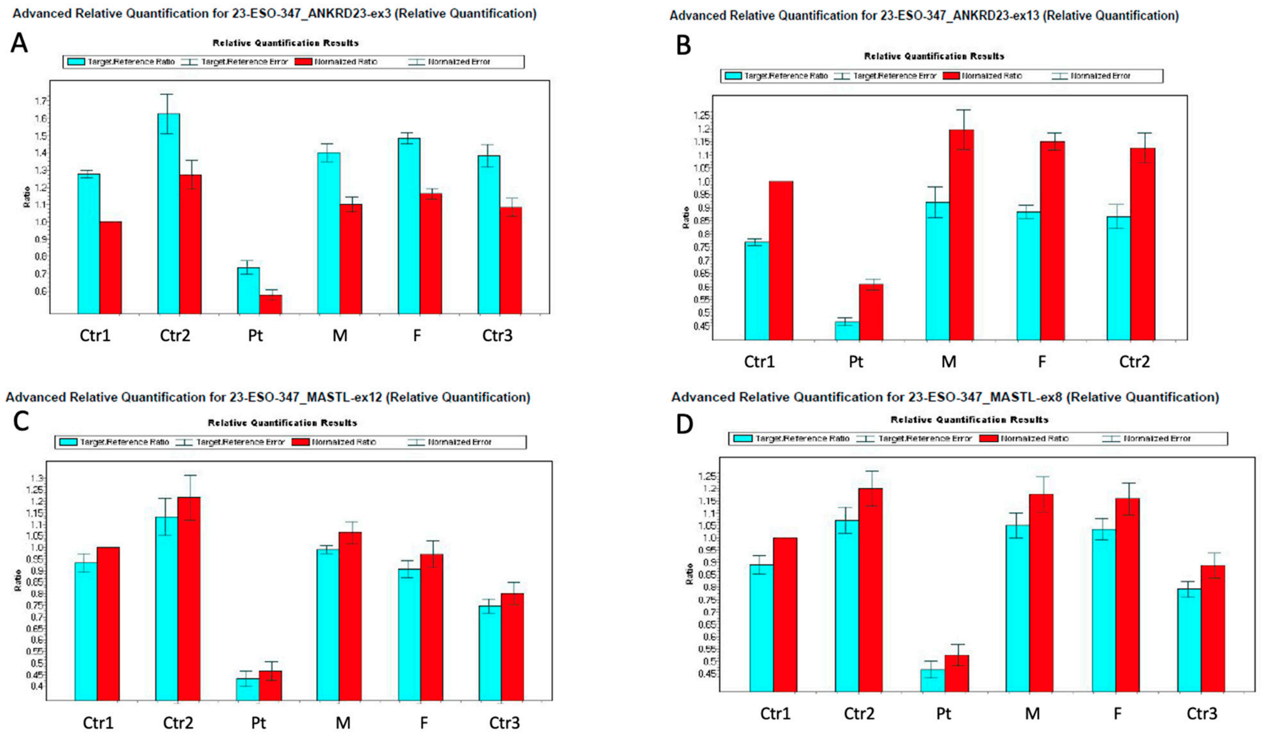

**Table S1.** Thrombocytopenia gene panel according to Human Phenotype Ontology.
*: coverage ≥99% with a target read depth 20X.

| | | | | | | |
|---|---|---|---|---|---|---|
| ABCG5 | ABCG8 | ACTB | ACTN1 | ANKRD26 | ARPC1B | CDC42 |
| CYCS | DIAPH1 | ETV6 | FLI1 | FLNA | FYB1 | GATA1 |
| GFI1B | GNE | GP1BA | GP1BB | GP9 | HOXA11 | IKZF5 |
| ITGA2B | ITGB3 | KDSR | MASTL | MECOM | MPL | MYH9 |
| NBEAL2 | PRKACG | PTPRJ | RBM8A | RUNX1 | SLFN14 | SRC |
| STIM1 | THPO | TPM4 | TRPM7 | TUBB1 | WAS | |

**Table S2.** Primers used in qPCR experiments

| Primer name | Primers (5′-3′) | Amplicon size (bp) |
|---|---|---|
| ANKRD26-ex3-F | AGGCTGTACAATGCCAGGA | 101 |
| ANKRD26-ex3-R | ATAGTGAAGAGCAGTGTTGCCA | |
| ANKRD26-ex13-F | TACCTTCTTGCATGAGTGGAT | 94 |
| ANKRD26-ex13-R | TCCATACCCATGTGGGCT | |
| MASTL-ex 8-F | GAACCATCCAGAATGAACATGA | 90 |
| MASTL-ex 8-R | AGTAGGGGTTATAGCCATGGGA | |
| MASTL-ex 12-F | TCCTAAGAGCTAAAACGTCATCCT | 105 |
| MASTL-ex 12-R | GGTATCTGTTTCATCATCTGGCT | |
| GAPDH-F | CTGCACCACCAACTGCTT | 87 |
| GAPDH-R | CCCAGCTCTCATACCATGAGT | |

**Table S3.** Primers used in Sanger Sequencing

| Primer name | Primers (5′-3′) | Amplicon size (bp) |
|---|---|---|
| mARPC1B ex5F | gttgtaaaacgacggccagtCAGTCGCCTCTCTCCTGG | 430 |
| mARPC1B ex5R | gtgtgaaattgttatccgctGAGGCCCATCCAGCTGA | |
